# Prognostic and predictive role of spatially positioned tumour infiltrating lymphocytes in metastatic *HER2* positive breast cancer treated with trastuzumab

**DOI:** 10.1038/s41598-017-18266-1

**Published:** 2017-12-21

**Authors:** Tiia J. Honkanen, Tiina Moilanen, Peeter Karihtala, Satu Tiainen, Päivi Auvinen, Juha P. Väyrynen, Markus Mäkinen, Jussi P. Koivunen

**Affiliations:** 1 Department of Oncology and Radiotherapy, Oulu University Hospital, Oulu, 90220 Finland; 2 University of Oulu, Oulu, 90014 Finland; 3 Medical Research Center Oulu, Oulu, 90014 Finland; 4 Cancer Center, Kuopio University Hospital, Kuopio, 70210 Finland; 5 University of Eastern Finland, Kuopio, 70211 Finland; 6 Cancer and Translational Medicine Research Unit, University of Oulu, Oulu, 90014 Finland; 7 Department of Pathology, Oulu University Hospital, Oulu, 90220 Finland

## Abstract

Disease outcomes of *HER2*+ breast cancers have dramatically changed after targeted therapies, such as trastuzumab, came to clinical practice but predictive factors for trastuzumab sensitivity and resistance are frequently unknown. Current work included metastatic breast cancer patients (n = 48), who were treated with trastuzumab and had pre-treatment tumour samples available. The tumours were immunostained for T-cell (CD3, CD8), natural killer (NK)-cell (CD56) and macrophage (CD68) markers and quantitative analysis of the immune cells was carried out using a computer-assisted image analysis in different tumour locations. High number of CD3 and CD8 positive T-cells was associated with significant survival benefit in the center of the tumour (CT) (p = 0.007, p = 0.001) but not in the invasive margin. The number of NK-cells and macrophages in the CT showed non-significant tendency towards improved survival. In subgroup analyses, high density of CD8 CT cells was associated with significant survival benefit in non-bone only disease, in TX or T1-3, and in ER+ tumours (p = 0.006, p = 0.003, p = 0.001). Moreover, high CD8 CT cell density associated significantly with long trastuzumab interruption periods in response. The results suggest important prognostic and predictive role of tumour infiltrating lymphocytes in center of the tumours in metastatic *HER2*+ breast cancer.

## Introduction

Immune system has suppressive and promoting effects on cancer development and progression, and both innate and adaptive immune cells are found in the tumour microenvironment^1^. Tumour infiltration of lymphocytes (particularly T-cells and natural killer (NK)-cells) have been associated with an improved survival rates in breast, colorectal, and gastric cancers^2–7^. Furthermore, tumour infiltrating immune cells have been linked to responses with chemotherapy and targeted cancer therapies^8–11^.

Human epidermal growth factor receptor 2 (*HER2*) gene is amplified in 20% of breast cancers and is associated with significantly more aggressive disease course including increased recurrence rate in localized disease and shortened overall survival in metastatic disease^12^. The development of HER2 targeted therapies, especially antibodies such as trastuzumab, has improved the patient outcome in this subset^13–17^.

Trastuzumab mode-of-action is related to both inhibition of oncogenic signalling and stimulation of antibody-dependent cellular cytotoxicity (ADCC)^14,18^. ADCC is mediated via activating Fc receptors (FcRs) in immune cells. Antitumour activity of trastuzumab was significantly reduced in mice deficient for FcRs, which demonstrates the critical role of ADCC for the activity of trastuzumab^19,20^. Fc receptors are expressed on the surface of NK-cells, natural killer T (NKT) -cells, macrophages and other components of the immune system^21,22^. Trastuzumab responses have been associated with increased tumour infiltration of NK-cells after drug administration. In addition, the infiltration of other immune cells, such as T- and B-cells, have also shown to be correlated not only with the trastuzumab efficiency but also with improved survival rates in *HER2*+ breast cancer^5,10,18,23–25^. However, more accurate, quantitative data on the significance of different immune cell types in different tumour locations would be required to reliably identify potential predictive markers for trastuzumab therapy.

In this current work, we examined the possible prognostic and predictive value of tumour infiltrating lymphocytes in metastatic *HER2* positive breast cancers treated with trastuzumab. We used a computer-assisted method to count the immune cells from the samples. Using this method gives many advantages, such as accuracy, reproducibility, objectivity and time efficiency compared to manual counting methods^2,26^.

## Results

### Patient characteristics

As a result of a systematic search we identified 54 patients, who had received intravenous trastuzumab in 2009–2014 for treatment of breast cancer and had metastatic disease at the time of diagnosis or relapsed disease not suitable for curative treatment. Of these patients, pre-chemotherapy samples were available for 48 (88.9%) patients and further analyses were limited to this group. Of the 48 samples, 31 (64.6%) were surgical and 17 (35.4%) were biopsy specimens. Furthermore, 14 (29.2%) of the patients were identified to have undergone trastuzumab treatment interruption in a tumour response.

### Image analysis of T-cells, NK-cells, macrophages and TLS

**Figure 1:**
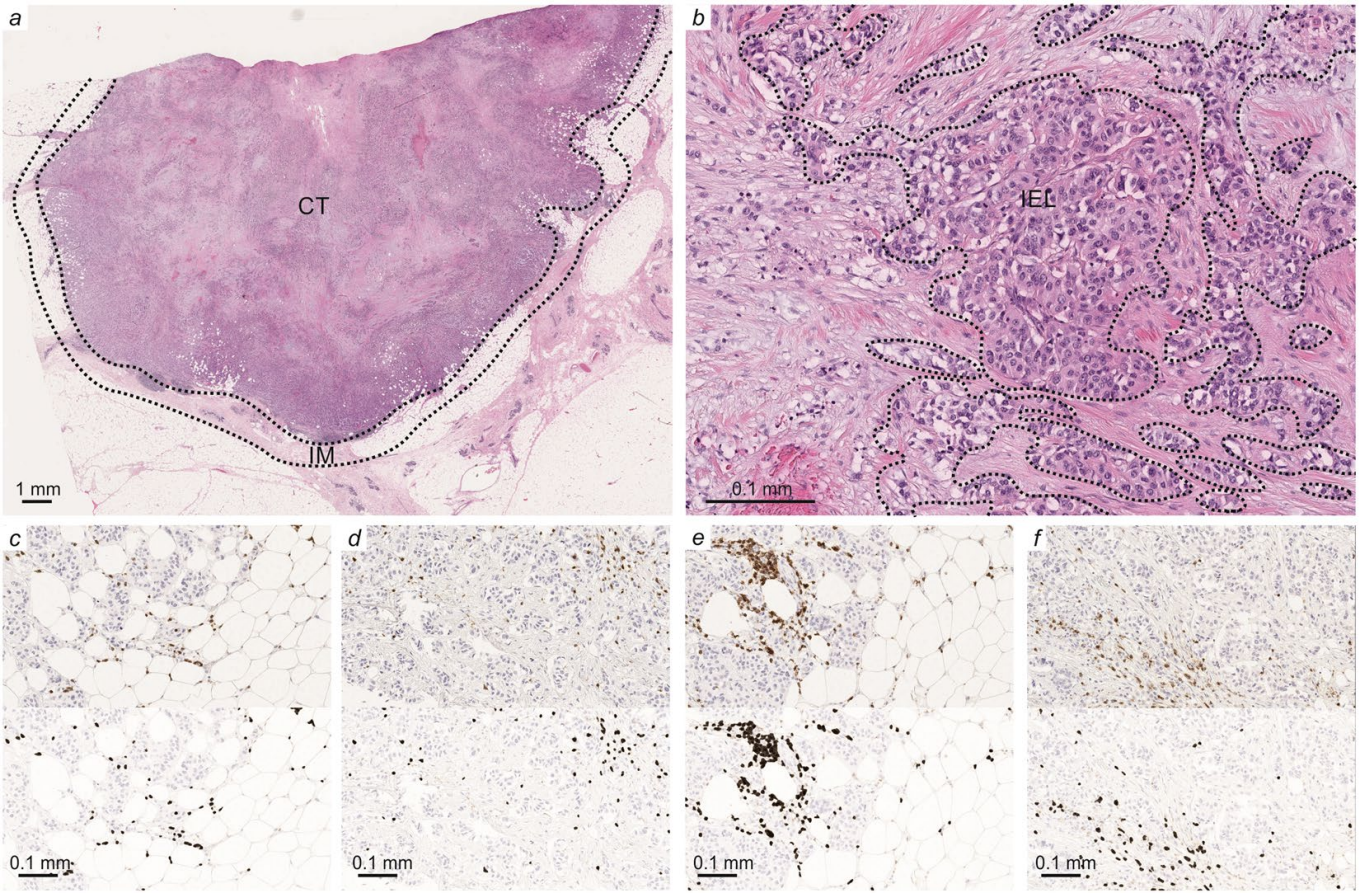
Immune cell infiltration in different tumour locations and result images of the image analysis. Different tumour locations in breast cancer: (**a**) Intratumoural (CT: center of tumour) and peritumoural (IM: invasive margin) location; (**b**) intratumoural area with circled intraepithelial (IEL) locations. (**c**–**f**) Immunohistochemistry images with the original captured picture as upper image and the counted cells are marked with dark grey shading in the lower image. (**c**) IM T-cells with CD3 staining. (**d**) CT T-cells with CD3 staining. (**e**) IM T-cells with CD8 staining. (**f**) CT T-cells with CD8 staining.

The tumours were stained for CD3 and CD8, which are general markers of T-cells (Fig. 1). While CD3 proteins are part of the T-cell receptor complex and found on the surface of every T-cell, CD8 co-receptors are found only on the surface of cytotoxic T-cells^27^. NK cells and macrophages were studied with CD56 and CD68 markers respectively. The quantitative analysis of all markers was successful from both surgical and biopsy specimens. However, CD56 and CD68 stainings were performed only for 40 patients due to lack of tumour material at the time of analysis. Furthermore, diameter and density of tertiary lymphoid structures (TLS) could not be analysed from biopsy specimens and therefore TLS were studied only from the surgical tumour samples (n = 31).

### T-cell, NK-cell and macrophage infiltrates and lymphoid follicle density and diameter

CD3 T-cells were most numerous in the invasive margin (IM) of the tumours, while in the center of the tumours (CT) and intraepithelial (IEL) locations these cells were less frequent. Similar findings were observed with CD8 T-cells (Table 1). The quantity of NK-cells was low in CT and IM, whereas macrophage infiltration was rather high in both locations (Table 1).

**Table 1:** Densities and ROC cutoff values of immune cells and lymphoid follicles.

		Median density (cells/mm^2^)	Range (cells/mm^2^)	ROC cutoff value (cells/mm^2^)
CD3	IM	573	19–2520	700
	CT	242	19–2033	300
	AVE	313	23–2207	300
	IEL	16	4–280	20
CD8	IM	207	20–1307	300
	CT	119	4–1200	120
	AVE	150	25–1236	230
	IEL	16	2–307	20
CD56	IM	14	2–140	17
	CT	9	1–114	12
CD68	IM	622	112–1465	646
	CT	666	319–1857	647
		**Median density (Lymphoid follicles/2x field, 95 mm** ^**2**^ **)**	**Range (Lymphoid follicles/2x field)**	**ROC cutoff value (Lymphoid follicles/2x field)**
TLS density		6.5	1–15	6.5
		Median diameter (mm)	Range (mm)	ROC cutoff value (mm)
TLS diameter		0.63	0.25–1.40	—

**Figure 2:**
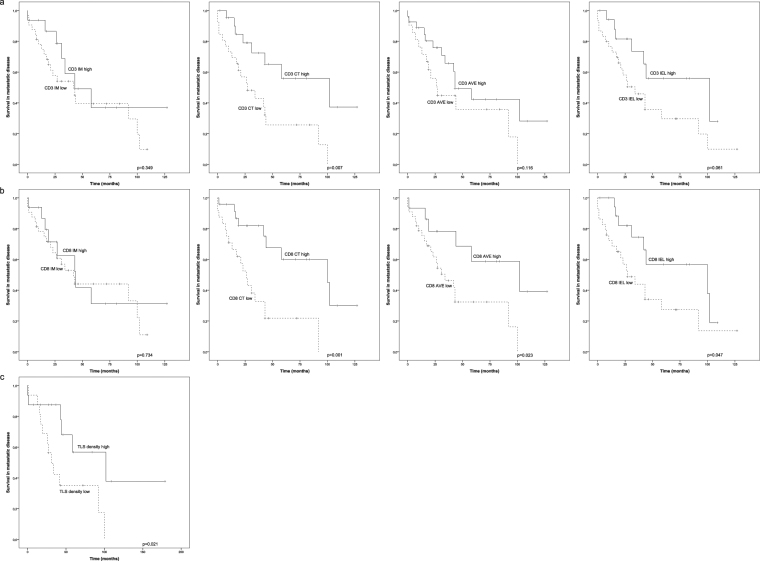
Survival analysis of metastatic *HER2*+ breast cancer in the presence of low or high T-cell infiltration. ROC analysis was used to set the optimal cutoff scores. (**a**) Kaplan-Meier estimates illustrating the association of CD3 stained T-cells with survival in IM, CT, AVE (IM + CT) and IEL locations (cutoffs 700, 300, 300 and 20 cells/mm^2^). (**b**) Kaplan-Meier estimates illustrating the association of CD8 stained T-cells with survival in IM, CT, AVE (IM + CT) and IEL locations (cutoffs 300, 120, 230 and 20 cells/mm^2^). (**c**) Kaplan-Meier estimates illustrating the association of TLS density (n = 31) with survival (cutoff 6.5 lymphoid follicles).

**Figure 3:**
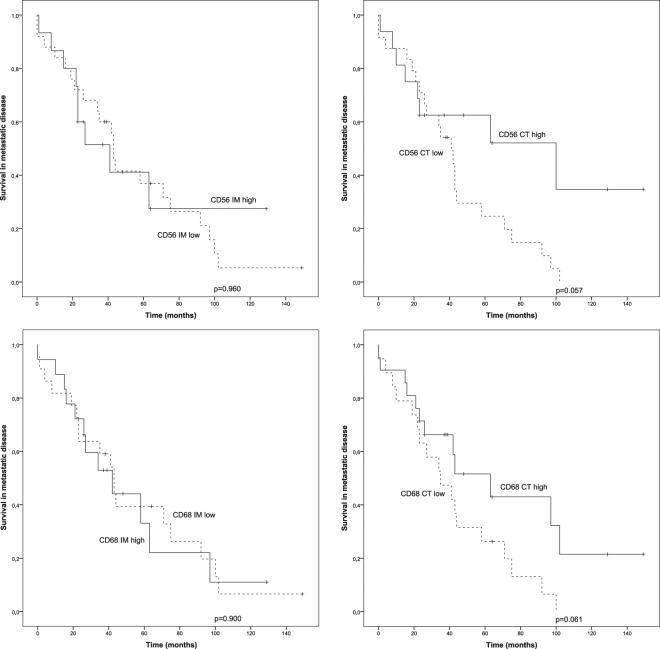
Survival analysis of metastatic *HER2*+ breast cancer in the presence of low or high NK-cell (CD56) or macrophage (CD68) infiltration. Kaplan-Meier estimates illustrate the association of NK-cells (upper row) or macrophages (lower row) with survival in the invasive margin (IM) and in the center of the tumour (CT). Cutoff values for CD56 IM, CD56 CT, CD68 IM and CD68 CT (17, 12, 646 and 647 cells/mm^2^) were set using ROC analysis.

For the survival analyses, the optimal cutoff scores for the immune cell densities were set using ROC analysis (Table 1), scores over cutoff representing high and scores under cutoff representing low density of stained immune cells. Kaplan-Meier survival estimates demonstrated an association between the density of CD3/CD8 cells and survival in metastatic disease (Fig. 2a,b). High number of CD3 T-cells in the CT area was found to be associated with a significantly improved survival (p = 0.007). Conversely, the high number of CD3 cells in the IM (p = 0.349), and IEL (p = 0.061) tumour locations did not show association with survival (Fig. 2a). Analogously to CD3, high number of CD8 positive cells in the CT area was associated with improved survival (p = 0.001). Furthermore, the density of CD8 positive cells associated with survival in IEL (p = 0.047) but not in IM (p = 0.743) areas (Fig. 2b). In addition, we analysed whether the average density of CD3 or CD8 T-cells in the whole tumour area (marked as AVE) had an association with survival. High average number of CD3 cells in the tumours did not show association (p = 0.116), whereas CD8 was found to be associated with improved survival (p = 0.023) (Fig. 2a,b). Survival analyses did not show any significant association between the NK-cell or macrophage densities and the survival. However, there was a tendency towards better survival in patients with higher CD56 (p = 0.057) or CD68 (p = 0.061) densities in the center of the tumour (Fig. 3).

We further analysed whether CD8 CT, the studied marker showing strongest association with prognosis, would remain as an independent prognostic factor in multivariate analysis. In the analysis, both CD8 CT density (HR 5.436, 95% CI 1.477–20.003; p = 0.011) and receiving trastuzumab as an adjuvant treatment (HR 40.136, 95% CI 4.472–363.473; p = 0.001) were independent prognostic factors when included in the same model. On the other hand, when *e.g*., ER status (HR 2.018, 95% CI 0.821–4.961; p = 0.126) and CD8 CT density (HR 4.621, 95% CI 1.839–11.608; p = 0.001) were included in the same model, only CD8 CT remained statistically significant.

We also analysed the tumours for the density and diameter of lymphoid follicles in the adjacent area of the tumour from H-E stained sections. The peak density of lymphoid follicles (TLS density, p = 0.021) was associated with improved survival (Fig. 2c), but the diameter of the largest lymphoid follicle had no association (not shown).

### Immunoscore

**Figure 4:**
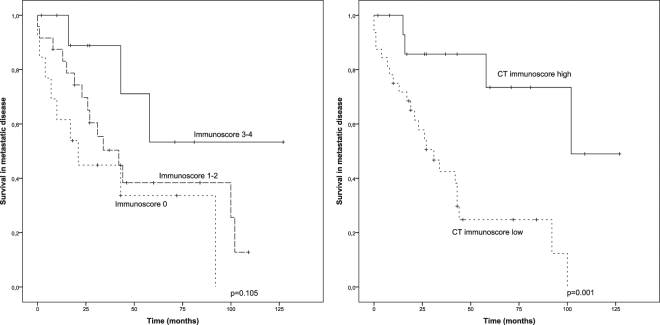
Survival analysis based on Immunoscore (CD3 + CD8). Kaplan-Meier estimates demonstrate the association of Immunoscore and CT Immunoscore with survival in metastatic disease. Immunoscore (CT + IM) is divided into 3 groups (0, 1–2, 3–4), where group 0 represents tumours with lowest densities of CD3 and CD8 and group 3–4 tumours with the highest densities. CT Immunoscore is divided into 2 groups (high, low) and tumours with high density of both CD3 and CD8 resulted as CT Immunoscore high.

Combined analysis of CD3 and CD8 cells in the IM and CT (Immunoscore) is a promising additional prognostic parameter in colorectal cancer, which categorizes the densities of each of these cell types in both IM and CT as low (0 points) or high (1 point), thus yielding a five-tiered classification (0–4). In colorectal cancer, high Immunoscore has been reported to predict improved survival better than either of the markers alone^28^. However, its significance in breast cancer is not well-known, and we carried out combined analysis of these markers in our material (CD3/CD8 density in IM and CT areas) and calculated the Immunoscore. Due to rather small number of patients in each of the Immunoscore groups, we combined the patients in three categories; 0 (n = 13), 1–2 (n = 24), 3–4 (n = 11). There was a tendency towards better survival in patients with higher Immunoscore but this, however, did not reach statistical significance (p = 0.105) (Fig. 4). Furthermore, we conducted the combined CD3 and CD8 analysis in the CT using two-class grouping; 0–1 low (n = 32), 2 high (n = 16). This classification (CT Immunoscore) associated significantly with survival (p = 0.001) but its prognostic value was not higher than seen with CD8 CT analysis alone (Fig. 4).

### Patient subgroups associated with CD8+ T-cell infiltrates

**Figure 5:**
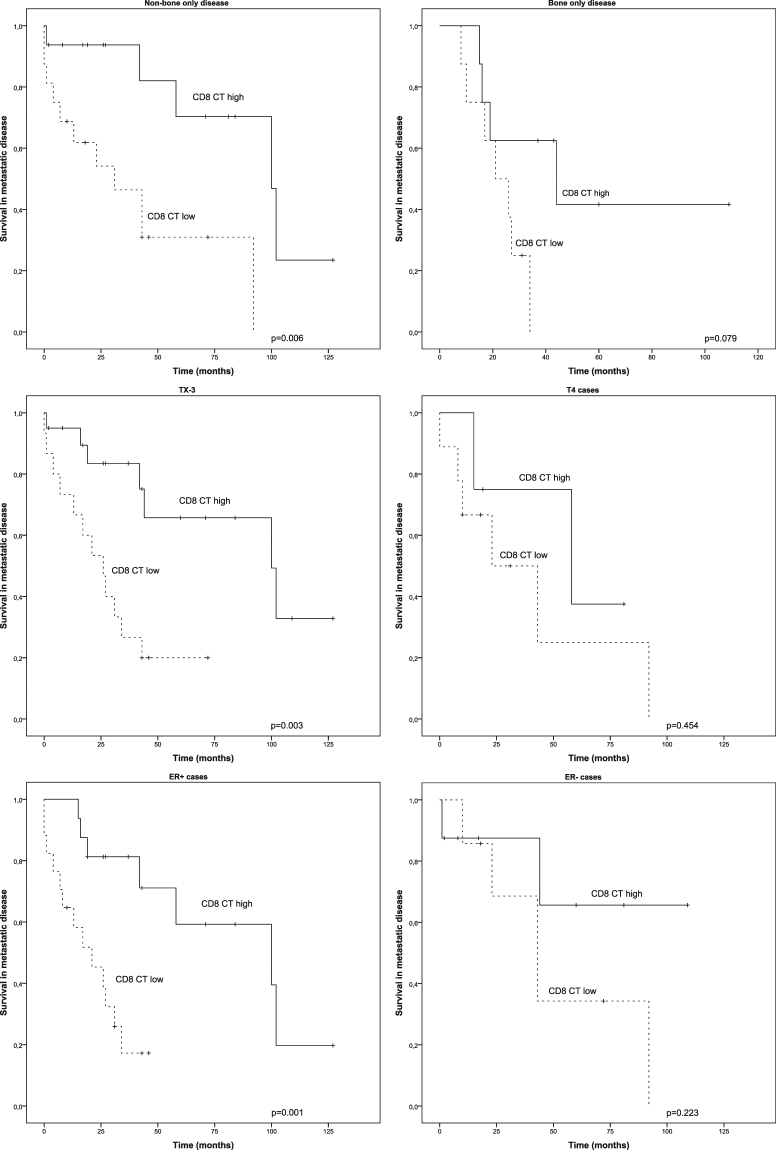
Survival analysis of patients in different clinical subsets according to CD8 center tumour status. Kaplan-Meier estimates illustrating association of CD8 CT cells and survival in non-bone- and bone-only disease, TX-3 (TX or T1-3) and T4 patients and ER+ and ER− patients.

We selected the density of CD8 positive T-cells in the CT area for further analysis in different patient subgroups, since this was identified as the best indicator for improved survival. The association between the high number of CD8 CT cells and survival in metastatic disease was seen in non-bone only disease (n = 32, p = 0.006) conversely to bone only disease (n = 16, p = 0.079). Furthermore, survival benefit association was seen in TX or T1-3 (n = 35, p = 0.003), but not in T4 tumours (n = 13, p = 0.454). The most significant association between the high number of CD8 cells in the CT and prolonged survival was found in ER positive subgroup (n = 33, p = 0.001) but not in ER negative cases (n = 15, p = 0.223) (Fig. 5).

### T-cell infiltrates and benefit from trastuzumab

**Figure 6:**
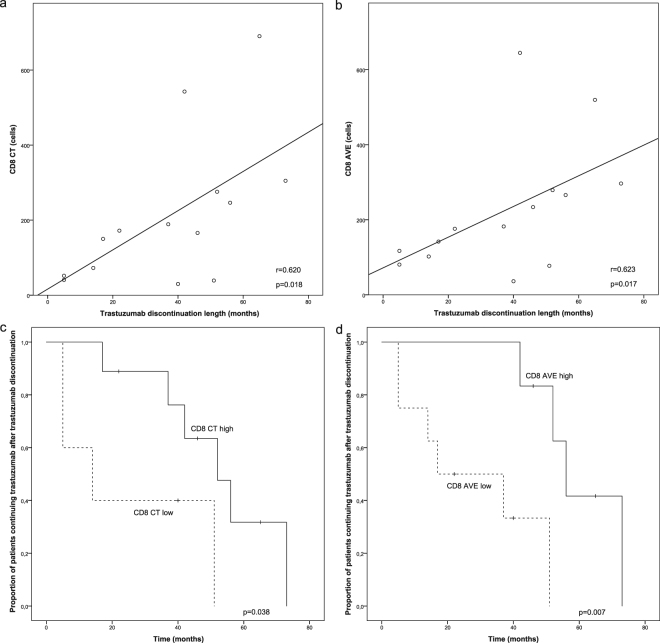
Correlation between CD8 status and the length of trastuzumab discontinuation. (**a**,**b**) Scatter plots are indicating the correlation between CD8 CT (r = 0.620, p = 0.018) or AVE (r = 0.623, p = 0.017) and the length of trastuzumab discontinuation. Spearman’s correlation was used to determine correlation coefficients (r) and p-values for each correlation. (**c**,**d**) Kaplan-Meier estimates for trastuzumab discontinuation length according to CD8 CT and CD8 AVE status.

Finally, we wanted to study whether the high number of CD8 CT cells could predict benefit from trastuzumab containing treatment in metastatic disease. We selected time from the beginning of 1^st^ line trastuzumab treatment to next line of treatment in metastatic disease (n = 43) or duration of longest trastuzumab interruption in response (n = 14) as indicators for trastuzumab benefit. We did not see any association between the 1^st^ line trastuzumab treatment length and any of the studied tumour immunological parameters. The length of trastuzumab interruption in response, however, correlated with CD8 IM (Spearman’s correlation coefficient (r_s_) = 0.541, p = 0.046, not shown), CD8 CT (r_s_ = 0.620, p = 0.018), and CD8 AVE (r_s_ = 0.623, p = 0.017) (Fig. 6a,b). Furthermore, trastuzumab discontinuation length associated with high CD8 CT and CD8 AVE patients (p = 0.038, p = 0.007) (Fig. 6c,d).

## Discussion

Disease outcomes of *HER2* positive breast cancer have changed dramatically after HER2 blocking antibodies, such as trastuzumab, became available. Trastuzumab mode-of-action is related to both target signalling inhibition and immune activation (ADCC) and therefore, both altered signalling pathways and immunological status of the tumour could be related to drug responses. Predictive factors for trastuzumab sensitivity and resistance are currently largely uncharacterized. Alterations abolishing the signal transduction inhibition of trastuzumab, such as p95 variant of HER2, PI3KCA, and PTEN, and compromised host immunoactivation have shown some correlation to trastuzumab resistance^29^. In this study, we asked whether high number of tumour infiltrating lymphocytes (TILs) could be used as prognostic and predictive markers in metastatic *HER2* positive breast cancer treated with trastuzumab.

ADCC is thought to be mainly mediated by NK-cell activation. Previous works with trastuzumab in *HER2* positive breast cancer have shown NK-cell infiltration and activation in tumours after drug administration and its relation to good response^5,25^. However, the prognostic and predictive role of NK-cells in pre-treatment tumour samples is mostly uncharacterized. Our results with pre-treatment tumour samples showed that the number of NK-cells was low and showed only a tendency towards better prognosis suggesting that NK-cell infiltration is not a good predictive marker for trastuzumab response in clinical tumour samples of *HER2* positive breast cancer. Our analysis was limited only to the pre-treatment tumour samples since only a few patients had post HER2-treatment samples available and these were after months of neo-adjuvant drug treatments.

A previous study has linked high number of pre-treatment TILs to good trastuzumab responses^10^. This study, however, only used morphological evaluation of the TILs in H-E stained sections. In our work, we analysed the density of CD3 (general T-cell marker) and CD8 (cytotoxic T-cells) positive T-cells by immunohistochemistry and computer-assisted image analysis in different tumour locations. This method enables more precise characterization of TIL location and their quantitative analysis^2,26^. Both of these markers (CD3 and CD8) and locations (CT, IM) are used to characterize the Immunoscore that is considered a promising prognostic parameter in colorectal cancer^2,28^. The results of our study showed that in *HER2* positive breast cancer, TILs were most prominently located in the IM than in the CT. Conversely to colorectal cancer, only TILs in the CT but not in the IM were associated with better prognosis. This may indicate that Immunoscore may not readily be adopted for different tumour types but needs to be fitted before the best possible prognostic value could be achieved. T-cells in the CT and IM might reflect different types of host immunoreaction to the tumour, but this requires further investigation. Of the studied markers, CD3 and CD8, the latter had significantly better prognostic value, supporting the importance of cytotoxic T-cells in tumour control. It still remains to be elucidated, whether TILs in *HER2* positive breast cancer are involved in the cytotoxicity of anticancer drugs directly or indirectly. Since cytokines are one of the mediators of immunity^30^, an interesting topic for future investigations could be how cytokine profiles affect the quantity of CD8 T-cells in the different tumour locations.

We also studied the association between CD8 T-cell infiltration and survival in different clinical setups. Prognostic value of high CD8 T-cell density was seen in non-bone only metastatic, ER positive and TX or T1-3 primary owning diseases but not in bone-only, ER negative and T4 disease. In former two of these groups, however, CD8 CT high phenotype showed tendency towards improved survival not reaching statistical difference, which could be related to the small sample size. Of the T4 tumours of our study, many presented as inflammatory breast cancers, frequently seen in *HER2* positive breast cancer, in which CD8 CT high phenotypes did not associate with survival. Inflammatory breast cancer, characterized by high angiogenesis and lymphangiogenesis^31,32^, may represent a cancer phenotype, which follows different laws of immunity.

A previous study of adjuvant trastuzumab in *HER2* positive breast cancer has shown that trastuzumab benefit limited only to patients with high TILs^10^ and therefore, we wanted to address whether CD8 CT phenotype could predict benefit from trastuzumab in metastatic setting. Retrospective tumour material of the study consisted of patients who all were treated with trastuzumab at varying points of their disease course, making the analysis more difficult. Based on evidence from randomized trials and personal experience, we selected treatment length of 1^st^ trastuzumab containing regimen in metastatic setting (surrogate of progression-free survival) and length of trastuzumab interruption in response as indicators for treatment benefit. In our institute, contrast to clinical trial protocols, we have aimed to discontinue trastuzumab treatment if a patient with metastatic *HER2* positive breast cancer has achieved prolonged radiological response and have seen that these patients can experience long trastuzumab-free treatment periods^33^. We were unable to see interaction between the length of trastuzumab therapy and the number of CD8 cells. However, in patients whose trastuzumab was discontinued in response, a positive correlation between trastuzumab discontinuation length and CD8 status was shown even though the number of studied cases (n = 14) was low. This suggests that trastuzumab therapy interruption in response could be feasible especially for CD8 CT high patients and should be investigated in prospective fashion. Patients with low CD8 CT counts have poor prognosis with current therapeutic options and molecular mechanisms behind the phenomenon should be further investigated.

In conclusion, this current study investigated prognostic and predictive role of tumour infiltrating lymphocytes in metastatic *HER2* positive breast cancer treated with trastuzumab using image analysis based quantitative method. The results suggest a significant role of high number of center tumour CD8 cells to better survival and benefit from trastuzumab therapy. Further prospective studies are needed to fully characterize the role CD8 in prognosis and therapeutic selection.

## Methods

### Patient data

All patients who had received at least one dose of intravenous trastuzumab at Oulu University Hospital in 2009–2014 were retrospectively identified from the pharmacy records. From electronic patient records, we identified those who had received trastuzumab for the treatment of metastatic breast cancer (n = 54) and excluded all those who had received the drug only as adjuvant treatment of breast cancer or as treatment for gastric cancer (n = 141). Study was limited only to those patients who had pre-chemotherapy tumour samples available (n = 48). *HER2* positivity was characterized by the presence of *HER2* amplification in chromogenic *in situ* hybridization. The patient data collection and tumour analysis were carried out under permits from the Northern Osthrobothnia Hospital District ethical committee (114/2011, amendment 23.02.2015), National Supervisory Authority for Welfare and Health (9850/05.01.00.06/2010), and the medical director of Oulu University Hospital (study no. 60/2015). According to national legislation of Finland, tissues gathered for diagnostic purposes can be used in scientific studies without an informed consent from the patient and therefore it was not obtained, and it is not relevant for the study. All the experiments were performed in accordance with relevant guidelines and regulations.

The patients’ age, date of diagnosis, date of metastatic disease, TNM staging, histological subtype, tumour grade, Ki-67 staining, estrogen receptor (ER) and progesterone receptor (PR) status, adjuvant/metastatic treatment regimens, treatment durations and therapy responses were collected from the electronic patient records. Survival in metastatic disease was calculated from the time of histological or radiological identification of metastatic disease to death or end of follow-up. Patients whose HER2 therapy was interrupted were characterized by having a planned HER2 therapy interruption in connection with a response lasting >12 months or with a response plus severe suspected HER2 therapy-related adverse events. HER2 therapy discontinuation length was defined from the date of the last administration of trastuzumab (in the longest HER2 therapy discontinuation period) to the date of drug reinitiation, death, or end of follow-up.

### Immunohistochemistry and immune cell counting

Immunohistochemistry was conducted on 3.5 µm sections cut from the paraffin-embedded specimens. The sections were deparaffinized in xylene and rehydrated through graded alcohols. Antigen retrieval was conducted in a microwave oven with Tris-EDTA (pH9) buffer at 800 W for 2 min and at 150 W for 15 min. The incubation with primary antibodies (CD3, Novocastra, Leica Biosystems, mouse monoclonal, clone PS1, dilution 1:50; or CD8, Novocastra, mouse monoclonal, clone 4B11, dilution 1:200; or CD56, Cell Marque, rabbit monoclonal, clone MRQ-42, dilution 1:100; or CD68, Dako, mouse monoclonal, clone PG-M1, dilution 1:100) was conducted at room temperature for 30 min. Bound antibodies were detected using the EnVisionTM (Dako) system. Diaminobenzidine (DAB) was used as the chromogen and hematoxylin as the counterstain.

For the analysis of the immune cell densities in different tumour locations (Fig. 1), sections were scanned with Aperio AT2 image-capturing device (Leica Biosystems). Imagescope (Aperio Technologies) software, version 11.2 was used to view the scanned images. With the software, 3–6 (median 6) images, depending of the size of the tumour sample, were captured with ×20 magnification from the center of the tumour (CT) and the invasive margin (IM). The immune cells were counted using an earlier described and validated computerassisted counting method^2,26^ that utilizes ImageJ, a freeware image analysis software^34^. Intraepithelial (IEL) immune cells were counted manually because the automated method was not able to distinguish the intraepithelial immune cells from stromal immune cells. Tertiary lymphoid structures (TLSs) were manually counted and measured from the H-E stained sections. Final counts presented in this manuscript are the average cell densities for each tumour area (immune cells), the diameter of the largest TLS in the samples (TLS diameter) and the highest density of TLSs/2x magnification (TLS density).

### Statistics

IBM SPSS Statistics 22.0.0.0 for Windows was applied for statistical analysis. The reported p-values are from two-sided chi-square tests. Receiver operating characteristics (ROC) analysis for the whole patient material was used to determine optimal cutoff scores of CD3, CD8, CD56, CD68 and TLS densities for discriminating the survivors from the non-survivors. Survival was analysed by using the Kaplan-Meier method with the log-rank test. Multivariate analysis was performed using Cox regression analysis. Correlation analysis was performed using Spearman’s correlation (2-tailed) and scatter plot analysis. Probability values below 0.05 were considered significant.

### Data availability

All data generated or analysed during this study are included in this published article.
